# Enhancing chemical synthesis planning: automated quantum mechanics-based regioselectivity prediction for C–H activation with directing groups

**DOI:** 10.3762/bjoc.21.94

**Published:** 2025-06-16

**Authors:** Julius Seumer, Nicolai Ree, Jan H Jensen

**Affiliations:** 1 Department of Chemistry, University of Copenhagen, Copenhagen, Denmark

**Keywords:** C–H activation, chemical synthesis planning, directing groups, quantum mechanics, regioselectivity prediction

## Abstract

The mild and selective functionalization of carbon–hydrogen (C–H) bonds remains a pivotal challenge in organic synthesis, crucial for developing complex molecular architectures in pharmaceuticals, polymers, and agrochemicals. Despite advancements in directing group (DG) methodologies and computational approaches, predicting accurate regioselectivity in C–H activation poses significant difficulties due to the diversity and complexity of organic compounds. This study introduces a novel quantum mechanics-based computational workflow tailored for the regioselective prediction of C–H activation in the presence of DGs. Utilizing (semi-empirical) quantum calculations hierarchically, the workflow efficiently predicts outcomes by considering concerted metallation deprotonation mechanisms mediated by common catalysts like Pd(OAc)_2_. Our methodology not only identifies potential activation sites but also addresses the limitations of existing models by including a broader range of directing groups and reaction conditions while maintaining moderate computational cost. Validation against a comprehensive dataset reveals that the workflow achieves high accuracy, significantly surpassing traditional models in both speed and predictive capability. This development promises substantial advancements in the design of new synthetic routes, offering rapid and reliable regioselectivity predictions that are essential for accelerating innovation in materials science and medicinal chemistry.

## Introduction

The activation and functionalization of carbon–hydrogen (C–H) bonds represent a fundamental challenge in modern organic chemistry, particularly because of the inherent stability and prevalence of these bonds in organic molecules. These bonds, which typically exhibit bond energies ranging from 90 to 110 kcal·mol^−1^, constitute the majority of bonds in organic chemicals. Therefore, their selective functionalization is essential for advancing the synthesis of complex molecules like pharmaceuticals, polymers, or agrochemicals [1–3].

Advancements in organometallic catalysis have facilitated significant progress in this area through C–H activation, transforming these inert bonds into reactive carbon–transition metal (C–M) bonds. Subsequent transformations of these complexes enable the formation of an array of new functional groups, such as carbon–carbon and carbon–heteroatom bonds, underpinning a plethora of synthetic applications.

Nevertheless, the high prevalence of C–H bonds in organic compounds presents a substantial challenge in achieving site-specific functionalization. A principal strategy to circumvent this challenge leverages directing groups (DGs) within the substrate, which coordinate to the metal centre of the catalyst, thereby dictating the site of C–H activation. Common DGs include unsaturated heteroatoms and alkenyl groups, which have proven effective in guiding the regioselectivity of these reactions [4].

**Figure 1:**
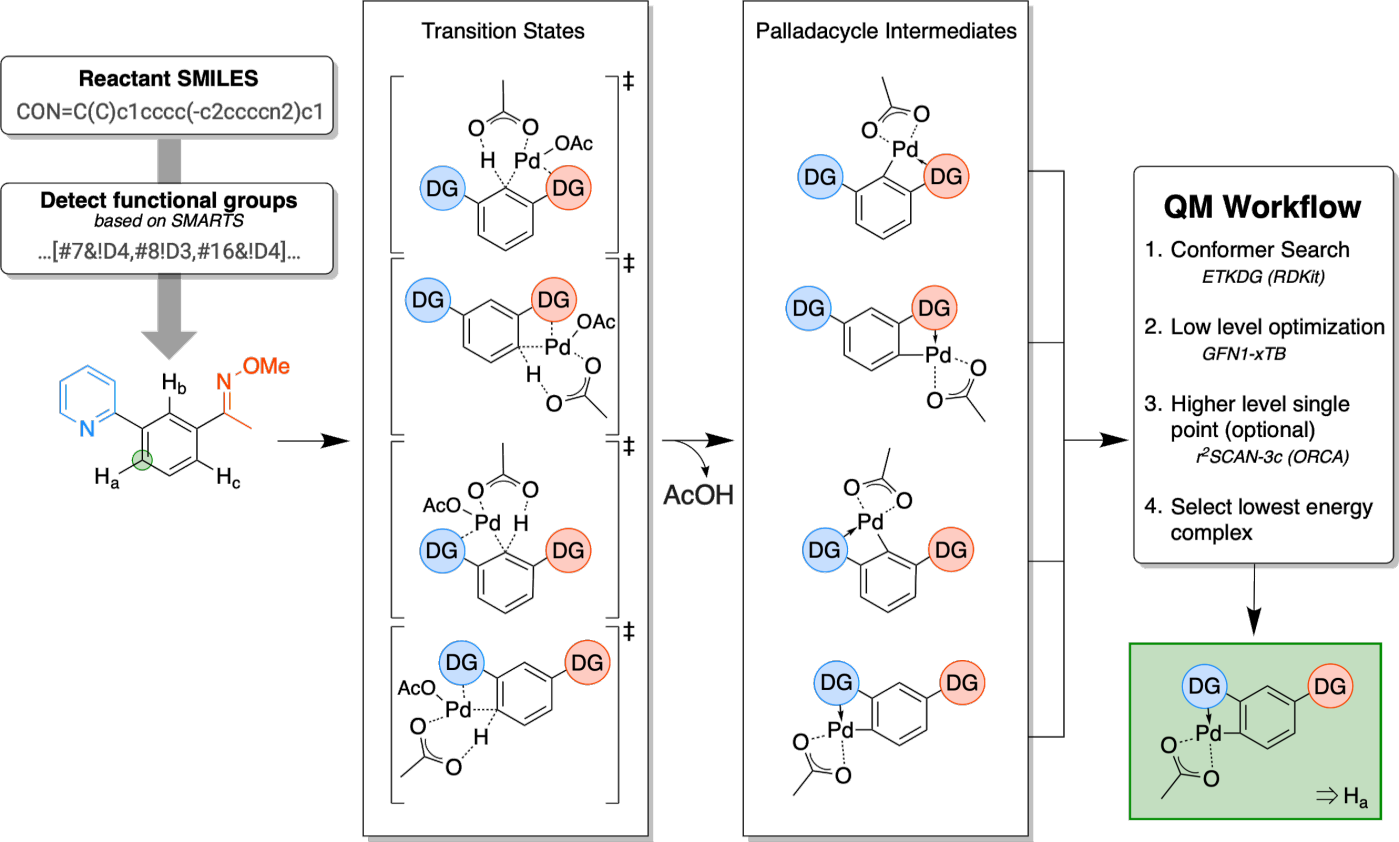
Overview of the predictive workflow: For the shown substrate on the left, three unique activation sites are possible (labelled “H_a−c_” with two directing groups, a pyridine (blue) and an oxime-ether (red) group. The latter has two potentially directing atoms, nitrogen and oxygen. The transition state structures of the rate-determining concerted metallation deprotonation (CMD) step are shown in the left column. In this work, we generate the structures of the proceeding palladacycle intermediate, shown in the right column. For each structure, we perform a conformer search followed by a low-level optimization (GFN1-xTB) followed by an optional higher-level single-point calculation (r^2^SCAN-3c). The lowest-energy complex is selected, and the corresponding reaction site is considered to be most likely to be activated, marked in green.

Mechanistic studies with palladium(II) acetate (Pd(OAc)_2_) as catalyst support the following mechanism of C–H activation, called concerted metal deprotonation (CMD) [5–7]. In a concerted mechanism, the Pd atom of the catalyst forms a sigma bond to an aromatic carbon, which increases the acidity of the adjacent (alpha) proton. This allows for the simultaneous abstraction of this proton by a carboxylate ligand. A directing group facilitates this step as it stabilizes the complex through coordination to the Pd atom, thereby lowering the reaction barrier. A depiction of the CMD step is shown in Figure 1.

Upon C–H bond breaking, the Pd atom moves into the plane of the aromatic ring, forming a palladacycle intermediate and carboxylic acid. The palladacycle intermediate can undergo further (coupling) reactions and form a variety of products via reductive elimination. In previous studies, the rate- and regioselectivity-controlling step was identified as the formation of the palladacycle [5–7]. The regioselectivity could be correctly predicted by calculation and comparison of the activation barrier of this step by Davies and colleagues [8]. Hence, the reaction site for which the activation barrier is the lowest is predicted to be the most probable one. Tomberg et al. [9] established that the regioselectivity could be predicted by calculation and comparison of the relative energies of the proceeding palladacycle intermediate, as postulated in the Bell–Evans–Polanyi (BEP) principle [10–11]. Focussing on the intermediates allows for easier automation of the calculations since a minimum instead of a saddle point structure on the potential energy surface is located, which can be done straightforwardly using standard optimization algorithms.

While the use of intermediate energies provides a computationally efficient alternative to explicit transition state searches, it rests on the assumption that there is a meaningful correlation between thermodynamic stability and kinetic accessibility, as expressed by the BEP principle. Although this principle has been successfully applied in many cases, the correlation between reaction energies and activation barriers is often imperfect. For instance, studies on hydrogen atom transfer and cycloaddition reactions have reported correlation coefficients (*R*^2^) of around 0.7 at best, indicating significant deviations from ideal behaviour [12–13]. This means that even when intermediate energies are accurately computed, the predicted regioselectivity may still carry a degree of uncertainty, which needs to be considered when applying BEP-based models to complex systems.

Tomberg et al. [9] introduced a hierarchy of directing strength for 238 different *ortho*-DGs, which can be used to rapidly predict the regioselectivity of C–H activation in complex molecules. The 238 directing groups are extracted from 150 molecules, taken from Chen et al. [4], for which reaction sites are known from experiments. For each directing group, the energy of the palladacycle intermediate with H-abstraction at a specific site is calculated using B3LYP-D3/LACVP** (6-31G**, except on heavy atoms where effective core potential was used) in CH_2_Cl_2_, and compiled into a hierarchical list for the determination of the reaction site with the lowest energy. Using the hierarchy, the regioselectivity of C–H activations could be rationalized for the 150 molecules with remarkable accuracy. While this approach performs well on this dataset, it does not generalize well to other molecules since not all relevant DGs are covered in the work by Tomberg and colleagues [9]. This is evidenced by our analysis using a dataset curated from Reaxys. Using our implementation of the method presented by Tomberg et al. [9] (for further details see section “Pattern matching”), we could only obtain correct predictions for four out of ten molecules, see section “Dataset curated from Reaxys”. This underscores the necessity for more robust and versatile predictive models that can adapt to the broad spectrum of organic chemistry’s structural variability.

Cao et al. [14] developed an automated workflow that predicts the regioselectivity of C–H activations using extensive DFT calculations on a HPC cluster using up to 600 nodes, each containing 16 Intel Xeon E5-2670 cores. They considered two possible reaction mechanisms, an electrophilic aromatic substitution and a proton abstraction mechanism via CMD, where they calculated the relative energies of the intermediates. Using their workflow, they were able to predict correctly the regioselectivity for 18 tested substrates. The main limitation of this work is the computational cost and usability since several DFT calculations need to be run on an HPC cluster in order to make a prediction.

In this study, we introduce a quantum mechanics (QM)-based computational workflow specifically developed to predict regioselectivity in C–H functionalization reactions involving directing groups following the CMD mechanism. This workflow employs (semi-empirical) quantum calculations in a hierarchical way to predict regioselective outcomes, delivering results within seconds to minutes. For substrates that are expected to follow the electrophilic aromatic substitution mechanism without the influence of DGs, we refer the reader to previous work done by Kromann et al. [15] and Ree and colleagues [16–17]. The there developed RegioSQM predicts the regioselectivity of reactions following the electrophilic aromatic substitution mechanism within seconds to minutes using a web interface or a Python module [18].

Similarly to previous works [9,14], we focus on the CMD step, the first and commonly the rate-determining step in C–H activation, and consider the prototypical Pd(OAc)_2_ catalyst. Using a selective approach, we calculate the relative energies of all relevant palladacycle intermediates using QM methods. We determine the relevant reaction sites either by a set of SMART patterns or by screening all possible reaction sites using the Merck molecular force field calculated ring strain energy, for details see section “Beyond *ortho*-directing groups”. This restriction allows us to rapidly predict the regioselectivity for C–H activations via the CMD mechanism within seconds to minutes on standard consumer hardware. The workflow accommodates various DGs and reaction conditions and can be extended to include not only *ortho* activations, as detailed in section “Results”.

This development holds the potential to significantly accelerate the discovery and optimization of new synthetic routes, thereby impacting materials science and medicinal chemistry by facilitating the synthesis of novel compounds with high precision and efficiency.

## Pattern Matching

**Figure 2:**
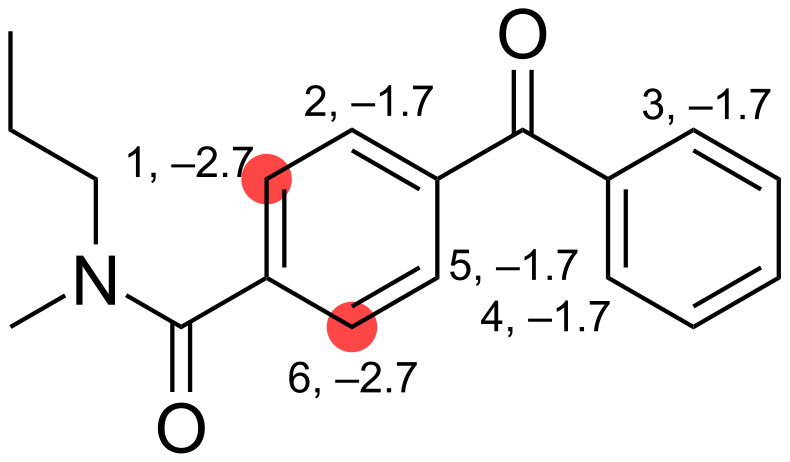
Example of the output from running the SMARTS pattern approach introduced by Tomberg et al. [9] with the predicted reaction site marked in red. All sites with a SMARTS pattern match are highlighted with Atom ID and DG strength in kcal·mol^−1^. For more details on the matched SMARTS patterns see Table 1.

Tomberg et al. [9] assembled a look-up table with 238 SMARTS patterns to compare the relative strength of different DGs and to determine which DG would yield the major product. This look-up table is convenient when dealing with a few molecules, but it quickly becomes cumbersome when examining lots of molecules. Thus, to make the work of Tomberg et al. [9] more accessible, we implemented a simple program that given a SMILES string goes through all of the SMARTS patterns to find matches, sorts the matches, and returns a visual output of the result. The algorithm sequentially sorts according to the number of heavy atom matches, the sum of atomic numbers of the heavy atom matches, and the DG strength associated with the SMARTS pattern. Hence, it favours SMARTS patterns that are most specific in terms of most atom matches, patterns with atoms having higher atomic numbers (matching a nitrogen atom is given a higher priority than matching a carbon atom), and the lowest DG strength if the other two priorities are unambiguous. Figure 2 and Table 1 show examples of the results from running the program with “CCCN(C)C(=O)c1ccc(C(=O)c2ccccc2)cc1” as the input SMILES string. The sites marked with red indicate the predicted reaction sites.

**Table 1:** Details on the results shown in Figure 2. The matched SMARTS patterns are sequentially sorted according to the number of heavy atom matches, the sum of atomic numbers of the heavy atom matches, and the DG strength associated with the SMARTS pattern.

Atom ID	SMARTS	Number of heavy atoms	Sum of atomic numbers	DG strength [kcal·mol^−1^]

1	[cH1]cC(N(C)A)=O	7	45	−2.7
1	[cH1]cC(N([C,c])[C,c])=O	7	45	−2.5
1	[cH1]cCN(C)C	6	37	−14.3
2	[cH1]c-C(=O)c	5	32	−1.7
3	[cH1]c-C(=O)c	5	32	−1.7
4	[cH1]c-C(=O)c	5	32	−1.7
5	[cH1]c-C(=O)c	5	32	−1.7
6	[cH1]cC(N(C)A)=O	7	45	−2.7
6	[cH1]cC(N([C,c])[C,c])=O	7	45	−2.5
6	[cH1]cCN(C)C	6	37	−14.3

The advantage of this pattern matching approach is that the method is extremely fast, as high-level QM calculations on structurally similar molecules are precalculated and stored in a database. However, the method also has some pitfalls, like how much structural information is needed in the SMARTS pattern to ensure that patterns are general enough to match new molecules but specific enough to only match DGs with similar DG strength. A choice that indeed affects how the SMARTS patterns should be prioritized if several of them match the same DG or reaction site.

**Figure 3:**
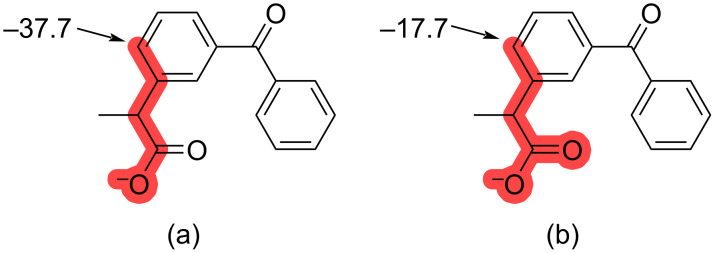
An example where our algorithm found a more specific SMARTS pattern match than highlighted in Tomberg and colleagues [9]. The matching SMARTS pattern from Tomberg et al. [9] is shown in (a), whereas our algorithm resulted in the match shown in (b).

As previously described, we decided to sequentially sort the matched SMARTS patterns according to the number of heavy atom matches, the sum of atomic numbers of the heavy atom matches, and the DG strength associated with the SMARTS pattern. This gave results that were in line with Tomberg et al. [9], although, for a few examples our algorithm resulted in more specific SMARTS patterns matching the DG. For example, Figure 3a shows the result from Tomberg et al. [9], which does not include the carbonyl oxygen in the SMARTS pattern match although the pattern marked in red in Figure 3b is part of the database. This example also highlights that in some cases the QM results are quite sensitive towards small structural changes.

**Figure 4:**
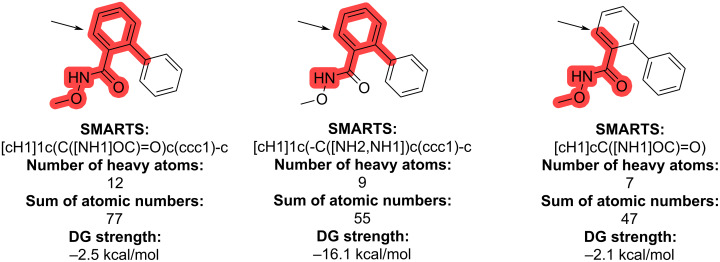
An example highlighting the difficulties in prioritizing the SMARTS patterns. All three patterns match the same site marked with the black arrow.

Another example that highlights the difficulties in prioritizing the SMARTS patterns is shown in Figure 4. The site marked with an arrow has three matching SMARTS patterns, which are sorted from left to right in accordance with our priority rules. The first two patterns result in quite different DG strengths, whereas the first and last patterns have similar DG strengths. In this case, the first pattern is really important as the assigned DG strength would otherwise have been completely different than intended.

## Computational Methodology of the QM Workflow

**Figure 5:**
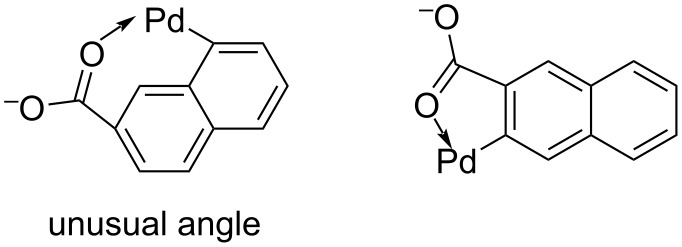
Example of a combination of C–H bond and DG that is discarded because of the angle constraint on the left and a combination that is considered valid on the right.

**Figure 6:**
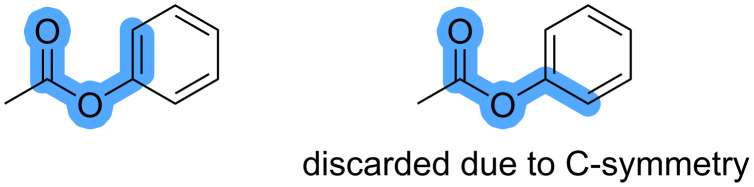
Example of combinations of C–H bonds and DGs that are considered identical because of symmetry of the C–H bond.

**Figure 7:**
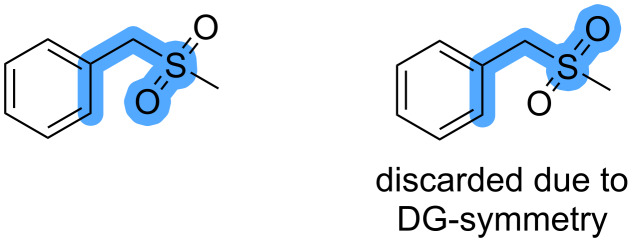
Example of combinations of C–H bonds and DGs that are considered identical because of symmetry of the DG.

**Figure 8:**
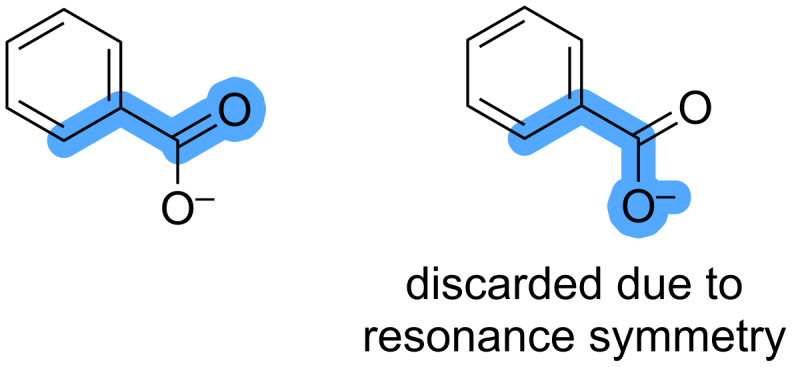
Example of combinations of C–H bonds and DGs that are considered identical because of resonance structures of the DG.

The here developed predictive QM-based model calculates which potential reaction site is most likely to react based on its corresponding reaction energy. Following the BEP principle, the relative energy of the intermediate should correlate linearly with the energy of the transition state [10–11]. The site with the lowest reaction energy is expected to correspond to the experimentally observed reaction site. Therefore, instead of locating the structure of the transition state, the preceding palladacycle intermediate structure is generated and optimized, as shown in Figure 1. Using this approximation the generation and optimization of structures simplifies greatly. In an automatized workflow, all unique and possible combinations of C–H bonds and *ortho*-directing groups (heteroatom with lone pair) in the substrate are found following this procedure:

1. All combinations of C–H bonds from sp^2^-hybridized C atoms and directing groups (heteroatom with lone pair) in the substrate, which are between two and five bonds apart from each other, are detected with SMART patterns. These patterns are general enough to cover all directing groups that were encountered in the literature sample from Chen and colleagues [4].

2. Next, we identify all relevant palladacycle complexes involved in the C–H activation facilitated by *ortho*-directing groups. For each match, a model complex is constructed containing the substrate and a Pd atom. In this complex, the Pd atom is bonded to the carbon at the reaction site and to the heteroatom of the directing group, as illustrated in Figure 5. To assess the geometry of these complexes, we generate a 2D structure using RDKit, where all atoms are constrained to lie in a single plane. Although this type of 2D embedding is typically used only for visualisation, it provides a quick way to screen for unrealistic geometries. We then measure the internal bond angles within the ring formed by the Pd atom, the directing group’s heteroatom, and the reactive carbon atom in the 2D structure. If any of these angles deviates by more than 10% from the ideal planar angle expected for a ring of *N*_atoms_ atoms, the match is discarded. The ideal angle is given by [(*N*_atoms_ − 2)·180°]/*N*_atoms_. This allows us to filter out complexes with strained geometries, such as the one shown on the left in Figure 5, using a simple approach.

3. Duplicate matches are removed when the reaction site is symmetric (Figure 6). Symmetry-equivalent sites are determined by comparison of the canonical SMILES for the substrate with an explicit hydrogen atom added to the corresponding reaction site. When two SMILES with an added explicit hydrogen at different atom indices are identical, then the corresponding atoms are symmetry-equivalent.

4. Duplicates are removed when the directing group is symmetric (Figure 7). Again, symmetry-equivalent atoms are determined by comparison of SMILES strings. Here, a bond to a dummy atom is added to the heteroatom of the directing group, and the canonical SMILES representation is compared to all other SMILES with an added dummy atom.

5. Duplicates are removed when the directing group has equivalent resonance forms, as shown in Figure 8. The equivalent heteroatoms are detected using SMARTS patterns for nitro and carboxylate groups.

For the remaining combinations of C–H bonds and directing groups, the corresponding intermediate substrate–Pd(OAc)–complex is generated. For each complex 3*N*_rot_ + 3 conformers are generated with ETKDG; here *N*_rot_ is the number of rotatable bonds in the substrate [19–20]. The conformers are clustered based on their RMSD with a cutoff of 1.0 Å, and the conformer corresponding to the centroid of each cluster is retained. The remaining conformers of each complex are optimized using GFN1-xTB in the implicit solvent model ALPB with parameters for CH_2_Cl_2_[21–22].

After each optimization, the geometry of the complex is analysed to determine whether the connectivity has changed. The connectivity of the complex before and after optimization is compared only for bonds not involving the transition metal since the determination of bonds to transition metals is prone to errors. Instead, the geometry of the four atoms adjacent to the transition metal (the two oxygen atoms from the acetate moiety, the carbon atom from the reaction site, and the heteroatom from the directing group) is analysed without regard for connectivity. All four atoms have to lie within a plane after the optimization for the optimization to be considered successful. This is determined by calculating the angle between the normal vectors of the plane spanned by Pd, the reaction site and the heteroatom of the directing group and the plane spanned by Pd and the two oxygen atoms of the acetate moiety. This angle has to be below 5° for the atoms to be considered to be within a plane.

Once all calculations for all conformers of all complexes are completed, the complex with the overall lowest-energy conformer is selected, and its corresponding reaction site is considered the most likely to react. All complexes that have conformers within a defined energy threshold of the overall lowest-energy conformer are considered to correspond to potential reaction sites; for this study, we choose a threshold of 1 kcal·mol^−1^.

When several complexes with conformers within the energy threshold are found and the corresponding reaction site differs between the complexes, we allow the user to refine the prediction by running r^2^SCAN-3c single-point calculations on the lowest-energy conformer of each complex within the energy threshold using ORCA [23–24]. This allows us to refine the predicted binding sites at a higher level of theory when this is required.

## Results

In the following, we tested our method on the dataset from Tomberg et al. [9] as well as on a new dataset that was curated from Reaxys [25]. In the evaluation, we considered the three categories “correct”, “semi-correct”, and “incorrect”. When the experimentally observed reaction site is the only reaction site that is predicted within the energy cutoff, the prediction is considered correct. When the experimentally observed reaction site is not the only reaction site that is predicted within the energy cutoff, the prediction is considered semi-correct, since we cannot distinguish beyond what is considered the chemical accuracy. When the experimentally observed reaction site is not one of the predicted sites, the prediction is considered incorrect.

### Dataset from Tomberg et al.

We consider 142 molecules with their experimentally determined reaction site from Tomberg et al. [9], which were originally curated by Chen et al. [4]. We are excluding cyclization reactions for which the regioselectivity is not only determined by the activation energy to form the palladacycle intermediate but also by which site is accessible for the intramolecular cyclization.

**Figure 9:**
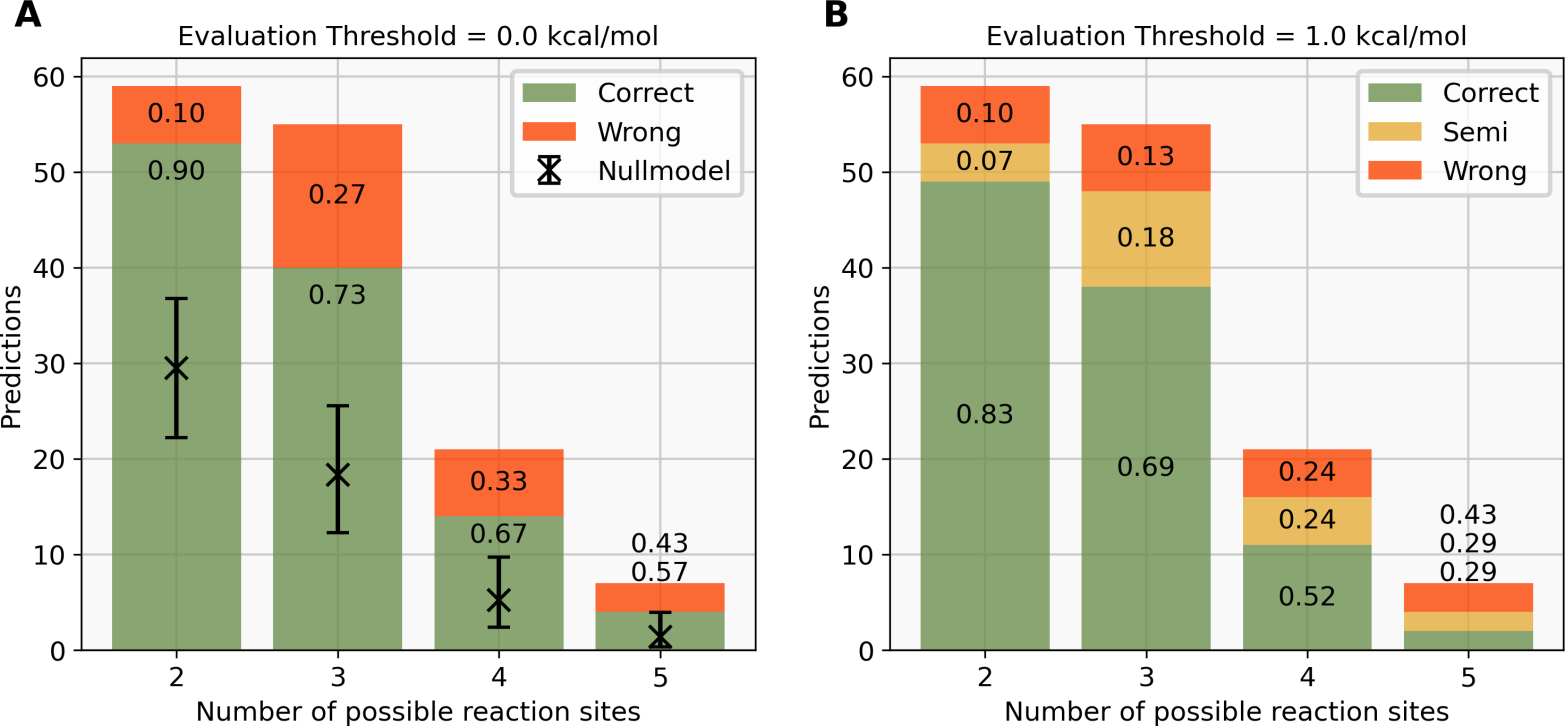
A: Distribution of correct (green) and wrong (red) predictions for molecules with two to five potential reaction sites, evaluated with an energy threshold of 0.0 kcal·mol^−1^. The numbers inside the bar plot correspond to the fraction of each label out of the total number of predictions. The expected performance of the null model with a 95% confidence interval is shown as a black cross. B: Distribution of correct (green), semi-correct (yellow), and wrong (red) predictions for the same molecules, evaluated with an energy threshold of 1.0 kcal·mol^−1^.

Using the previously described workflow, we were able to predict the experimentally observed reaction site with 78% accuracy over the whole dataset when using no energy threshold, meaning that only the reaction site corresponding to the lowest-energy complex is predicted to be the reaction centre. In Figure 9A, the predictions, correct (green) or wrong (red), are shown as a stacked bar chart for molecules with different numbers of potential reaction sites. The expected number of correct predictions and the 95% confidence interval of a model that guesses one of the potential reaction sites at random is shown as a black cross.

**Figure 10:**
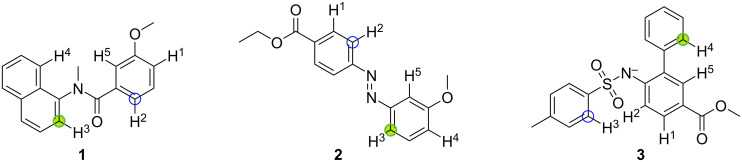
Molecules with five potential reaction sites that are predicted wrong by the QM workflow. The experimentally observed and computationally predicted reaction sites are marked by green and blue circles, respectively.

For molecules with only two potential reaction sites, the null model, which picks reaction sites randomly, is expected to correctly predict the reaction site for 30 out of 60 molecules. Our QM-based workflow can predict the correct reaction site for 54 out of 60 molecules with two potential reaction sites, which corresponds to 90% correct predictions and lies outside of the confidence interval of the null model. Similarly, for molecules with three and four potential reaction sites, the QM workflow predicts 73% and 67% of the reaction sites correctly, when we would expect the null model to guess the correct reaction site with an accuracy of 33% and 25%, respectively. Notably, the QM workflow predicts the correct reaction site for only four out of seven molecules with five potential reaction sites, which corresponds to 57% accuracy. The three molecules with five potential reaction sites and wrong predictions are shown in Figure 10 with the experimentally observed reaction site in green and the predicted reaction site marked by a blue circle.

For molecule **1**, we can see in the original paper from Yeung et al. [26] that the reaction preceding the C–H activation is an intramolecular cyclization between the C atom marked in green and the C atom marked by a blue circle. This reaction was originally not labelled as a cyclization reaction, which is why we did not remove it from the dataset. Nevertheless, upon inspection, our QM workflow correctly predicts the reaction site(s) of the intramolecular cyclization as it predicts one of the two reaction sites for the C–H activation.

The reaction site of molecule **2** from Dong et al. [27] cannot be predicted correctly as the experimentally observed reaction site is 1.7 kcal·mol^−1^ higher in energy than the predicted site at the r^2^SCAN-3c level. This would correspond to a ten times higher rate constant of the reaction leading to the other regioisomer at the reaction temperature of 90 °C. Experimentally, it is observed that the regioselective C–H activation happens on the more electron-rich aromatic ring with the methoxy substituent as opposed to the one with the alkoxycarbonyl group. The wrong prediction here might indicate that the BEP relationship does not hold in this case and one would need to calculate the activation energy of the actual transition states.

Molecule **3** from Jiang et al. [28] is another intramolecular cyclization reaction which was not labelled as such. For such reactions, the regioselectivity is not only determined by the activation energy for the rate-determining step but also by the proximity of an intramolecular reaction partner, here the secondary amine.

From this in-depth analysis, we conclude that our QM workflow only predicted the wrong reaction site for one out of these three molecules investigated as the other “incorrect” predictions are due to a problem with the underlying dataset.

Since we do not assume that the energies obtained at the r^2^SCAN-3c(CPCM)//GFN1-xTB(ALPB) level are accurate enough to separate regioisomers which are close in energy, we consider all reaction sites that are within a threshold of 1 kcal·mol^−1^ of the lowest-energy reaction site as potential reaction sites. This threshold was chosen based on the tradeoff between reducing the number of wrong predictions while simultaneously minimising the number of semi-correct predictions. The fraction of each prediction class (correct, semi-correct, wrong) as a function of the evaluation threshold is shown in Figure S1, Supporting Information File 1. Here, one can see that the number of wrong predictions is reduced by more than 30% when using an evaluation threshold of 1 kcal·mol^−1^ while the number of correct predictions only decreases by 10%. When more than one reaction site is within this threshold, we label the prediction as “semi-correct”. Depending on the use case, the user might want to proceed with optimizing the structures of the relevant complexes at a higher level of theory or perform a transition state search to calculate the activation energy. With this threshold, we obtain 70% correct, 14.5% semi-correct, and 14.5% wrong predictions over the whole dataset. For six out of 17 molecules, all possible reaction sites are predicted as reaction sites within the threshold as shown in Figure S7, Supporting Information File 1. This means that these predictions do not yield any information, but for the other cases, the prediction rules out other potential reaction sites.

We implemented the approach presented by Tomberg et al. [9] which predicts the reaction site based on pre-computed relative energies at DFT level of theory for a selection of C–H bonds that are identified using SMARTS patterns. Using this approach, we achieved an accuracy of 92% with a total runtime of less than one second.

### Dataset curated from Reaxys

From a query in Reaxys (see Supporting Information File 1, section “Reaxys Query for C–H activation”), we selected 10 C–H activation reactions with Pd(OAc)_2_ as the catalyst and multiple DGs and/or symmetry-nonequivalent reaction sites. Using our QM workflow, we were able to predict the regioselectivity of nine out of ten molecules (semi-)correctly. Five reaction sites were predicted to be within 1 kcal·mol^−1^ of another possible reaction site in the reactant and were therefore classified as semi-correct, meaning we cannot predict with our model which of the two regioisomers will be the main product of the reaction. Refinement using r^2^SCAN-3c single-point calculations did not result in better agreement with experimental observations.

**Figure 11:**
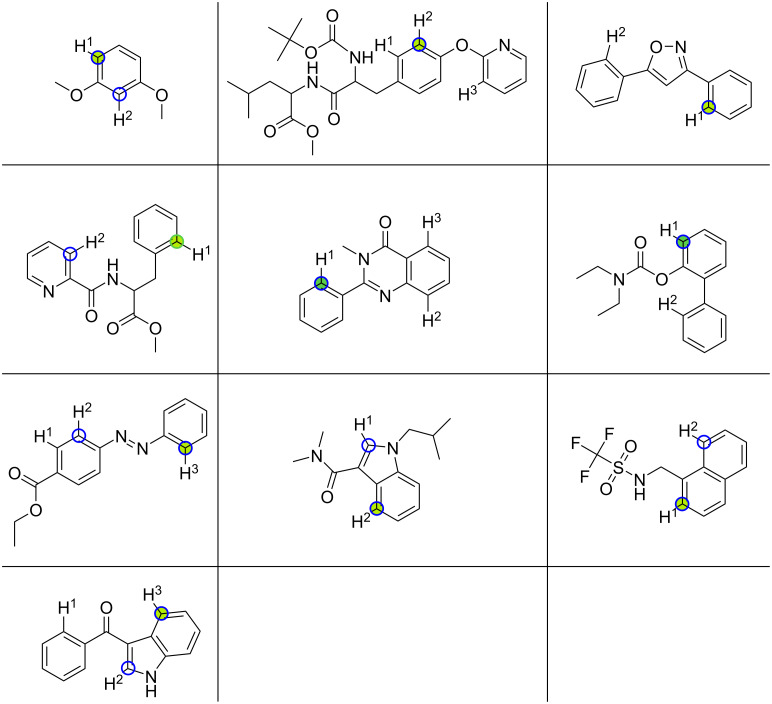
Predictions of reaction sites within a 1 kcal·mol^−1^ threshold for ten molecules are marked with a blue circle, and experimentally observed reaction sites are highlighted by a green circle.

The ten molecules with their experimentally observed main reaction site in green and all predicted reaction sites within a 1 kcal·mol^−1^ threshold as a blue circle are shown in Figure 11.

Using the previously mentioned approach following Tomberg et al. [9], we were able to obtain four out of ten correct predictions. This is slightly worse than a null model that guesses the reaction site at random, which would yield an accuracy of 0.46. This can be attributed to the fact that no data is available for several relevant C–H bonds in these ten molecules. For three out of the ten molecules, no pre-computed data for any of the C–H bonds was available, therefore, no prediction can be made.

### Beyond *ortho*-directing groups

**Figure 12:**
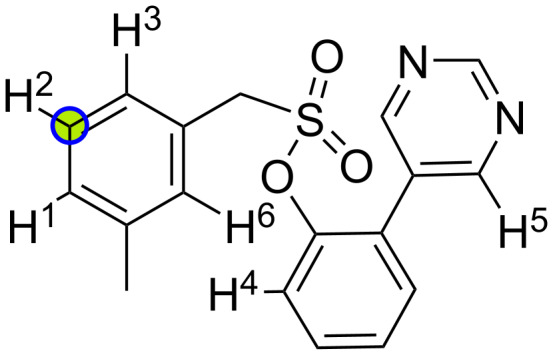
Substrate with six potential unique reaction sites for C–H functionalization. The experimentally determined reaction site is marked by a green circle, the computationally predicted one is marked by a blue circle.

Here we showcase how the workflow can be extended for the application of the workflow to a substrate with a *meta*-directing group. The substrate was investigated by Achar et al. [29], and the reaction site was determined by the authors to be the H^2^ with a *meta*/other regioselectivity of up to 25:1 and a yield up to 85% (Figure 12).

In order to extend our approach to *meta*-/*para*- and remote-directing groups, we use a different approach to identify relevant palladacycle complexes as in points 1. and 2. outlined in the section “Computational Methodology of the QM Workflow”. Instead of using SMARTS patterns to detect pairs of *ortho*-directing groups and reaction sites, we detect all potential reaction sites by detecting all C–H bonds at sp^2^-hybridized carbon atoms as well as all heteroatoms with lone pairs separately and remove symmetry-equivalent sites. Then, we obtain all potential pairs of C–H bonds and heteroatoms as the Cartesian product of the two sets. Next, we filter out all pairs for which no reasonable 3D geometry can be generated. To determine whether or not a pair of C–H bond and heteroatom can form a reasonable 3D geometry, we generate a 3D geometry of a dummy “palladacycle” intermediate between the substrate and a CCl_2_ fragment using ETKDG. The CCl_2_ fragment is used to mimic the Pd(OAc)_2_ catalyst, which cannot be used since the following step relies on the Merck molecular force field (MMFF, version MMFF94s), which is not parameterized for transition metals like Pd [30–31]. If the embedding fails, the corresponding pair is removed. When a 3D geometry could be obtained, we optimized the structure using the MMFF94s. Next, we calculate the sum of (out-of-plane) angle terms and torsion terms of the MMFF94s force field for the optimized structure. The geometry is considered reasonable if the sum of the angle and torsion terms is below a threshold of 10 kcal·mol^−1^. From here on, we proceed with the workflow as described in the section “Computational Methodology of the QM workflow”.

For the here considered substrate, this procedure reduces the number of complexes to optimize with GFN1-xTB from 30 to nine; the complexes are shown in Figure S2, Supporting Information File 1. This procedure involves several force-field optimizations, which increase the overall wall time by ≈10 s for the here shown substrate compared to the previously reported approach. From here on, we follow the same procedure as for the *ortho*-directing groups and correctly predict the reaction site H^2^, which is the only one within the 1 kcal·mol^−1^ energy threshold at the GFN1-xTB level.

## Discussion

Our study demonstrates that our fully automated QM-based workflow reliably predicts the reaction site as observed experimentally with 70% correct predictions and 14.5% semi-correct predictions on the dataset provided by Tomberg and colleagues [9]. Analysis of molecules where the reaction site was incorrectly predicted, particularly those with five potential sites, revealed that there might be issues with the underlying data in some cases. When only considering the lowest-energy reaction site predicted by our workflow, we were able to achieve an accuracy of 78% on the same dataset. In contrast, a null model making random guesses would achieve only 38% accuracy, with a 95% confidence interval from 36 to 40%, underscoring our workflow’s superior performance.

Additionally, we applied the workflow to a new set of ten molecules, achieving a 90% accuracy rate in predicting C–H activation sites. We also explored the tool’s capability to predict regioselectivity in C–H activation with various directing groups, not limited to *ortho*-directing groups. By identifying potential reaction site-directing group pairs using an approach based on MMFF energies instead of simple SMARTS patterns, we illustrated the workflow’s effectiveness with a case study from existing literature, accurately predicting the reaction site in a *meta*-directing C–H activation scenario.

Compared to previous work by Tomberg et al. [9], our workflow is considerably more compute-intensive to run, since (semi-empirical) QM calculations are performed. Yet, we are able to perform predictions on all molecules, not only on molecules for which there is pre-computed data for all the relevant C–H bonds. This is especially relevant when making predictions on molecules that were not part of the dataset used for the method development, as highlighted in the section “Dataset curated from Reaxys”. Even though data for all relevant C–H bonds from over 150 molecules were pre-computed, the resulting molecular patterns are very specific, and the approach does not generalize well.

In this study, we rely on several key assumptions that we will outline below. First, we focus exclusively on the regioselective outcomes of reactions using the concerted metallation deprotonation (CMD) mechanism between the catalyst and the substrate. It is important to note that this approach does not allow us to predict the occurrence of the reaction, its yield, or confirm if the reaction might proceed via a different mechanism influenced by the substrate, catalyst, and ligands. Second, we assume that the reaction is controlled kinetically, where the activation energy required to form the palladacycle intermediate determines the C–H activation regioselectivity. This assumption holds true primarily when the reaction is irreversible, and the formation of the intermediate is the rate-limiting step. While previous studies support this assumption, it may not always apply universally across various substrates or catalysts [8]. Third, we consider the linear energy relationship between the intermediate and its preceding transition state as per the Bell–Evans–Polanyi principle. However, this relationship may not provide sufficient accuracy for making predictions when the energy difference between reaction sites is less than 1 kcal·mol^−1^. To enhance the reliability of our predictions, ideally, we would automate the process of locating transition state structures.

To enable rapid predictions, ranging from seconds to minutes on consumer hardware, we employ semi-empirical optimizations and, when necessary, DFT single-point calculations to reduce computational costs. In our analysis using the dataset from Tomberg et al. [9], we recorded median and mean prediction times of 2:02 and 2:21 minutes, respectively, using four Intel Xeon E5-2643 v3 (3.4 GHz) CPUs. These times were significantly reduced to 22 and 34 s when exclusively using semi-empirical optimizations. The workflow benefits from parallelized QM programs and routines, demonstrating nearly linear reductions in wall time as the number of cores increases, tested up to 16 cores. However, for greater accuracy, particularly at reaction sites with energy differences less than 1 kcal·mol^−1^, DFT optimizations are recommended, as they may necessitate higher-level (re-)optimization for precise predictions.

The primary strength of the QM workflow lies in its flexibility, which facilitates customization through various means, such as simulating different solvent effects or examining the impact of different catalysts and ligands, extending beyond Pd(OAc)_2_. Additionally, accounting for varying reaction conditions, like conducting the reaction in acidic or basic environments, is possible by adjustments to the substrate-SMILES. Protonation states of substrates can be predicted using either machine learning models [32] or QM calculations [33].

This developed workflow is designed to be accessible not only to computational chemists, but also to those without a computational background, through multiple interfaces, including a command line interface, a web-based user interface, an API, and a stand-alone Python module for integration into more complex systems. For example, this workflow can be used in further molecular discovery and optimization to design specific directing groups that can facilitate the functionalization of remote C–H bonds, like *meta* or *para* functionalization. This can be done by using the workflow in the scoring function of a genetic algorithm, for example. Here, the absolute directing strength towards a specific site can be used to score different directing groups to each other and have the genetic algorithm design molecules that increase the directing strength of a directing group towards a specific site.

While this paper was in review, Oshiya and co-workers published an ML model that classifies reactants as having *ortho*-, *meta*-, *para*- or non-directing functionalities for Pd-catalysed directing group-assisted C–H activation [34].

## Supporting Information

File 1 Additional computational data.

## Data Availability

Code and data is available at https://github.com/jensengroup/regiotm.
