# Supplementary material for: Enhancing chemical synthesis planning: automated quantum mechanics-based regioselectivity prediction for C–H activation with directing groups
Source: Beilstein J Org Chem. 2025 Jun 16;21:1171–82. doi: 10.3762/bjoc.21.94 (PMC12207270; doi:10.3762/bjoc.21.94)
Supplement: File 1 — Additional computational data. [file Beilstein_J_Org_Chem-21-1171-s001.pdf]

BEILSTEIN JOURNAL OF ORGANIC CHEMISTRY

Supporting Information

for

Enhancing chemical synthesis planning: automated quantum mechanics-based regioselectivity prediction for C–H activation with directing groups

Julius Seumer, Nicolai Ree and Jan H. Jensen

Beilstein J. Org. Chem. **2025,** *21,* 1171–1182. doi:10.3762/bjoc.21.94

Additional computational data

License and Terms: This is a supporting information file under the terms of the Creative Commons Attribution License (https://creativecommons.org/licenses/by/4.0). Please note that the reuse, redistribution and reproduction in particular requires that the author(s) and source are credited and that individual graphics may be subject to special legal provisions.
The license is subject to the *Beilstein Journal of Organic Chemistry* terms and conditions: (https://www.beilstein-journals.org/bjoc/terms)

Reaxys query for C–H activation

The query on Reaxys consists of the following steps:

1. Search for “C–H activation”, which yields 553472 reactions.

2. Filter by catalyst “$Pd(OAc)_2$”, which yields 705 reactions.

3. Filter by reaction class “C–C bond formation”, which yields 17 reactions.

4. Selecting the one reaction that is actually a Pd-catalyzed C–H activation with a directing group and search for similar reactions with criterion “wide”, which yields 37105 reactions.

5. Filter by catalyst “$Pd(OAc)_2$” which yields 1955 reactions.

6. Filter by reaction class “C–C bond formation” and the subcategories “Ar-H to Ar-CH2-”, “ArH to Ar-C(=)-”, “ArH + -C(=)-X to Ar-C(=)-”, “ArH + -C(=)-O- to Ar-C(=)-”, and “ArH + -CH2-O- to Ar-CH2-”, which yields 330 reactions.

7. Out of the 330 reactions, we chose all examples that had several potential reaction sites and/or directing groups, which yields 10 reactions.

Evaluation threshold

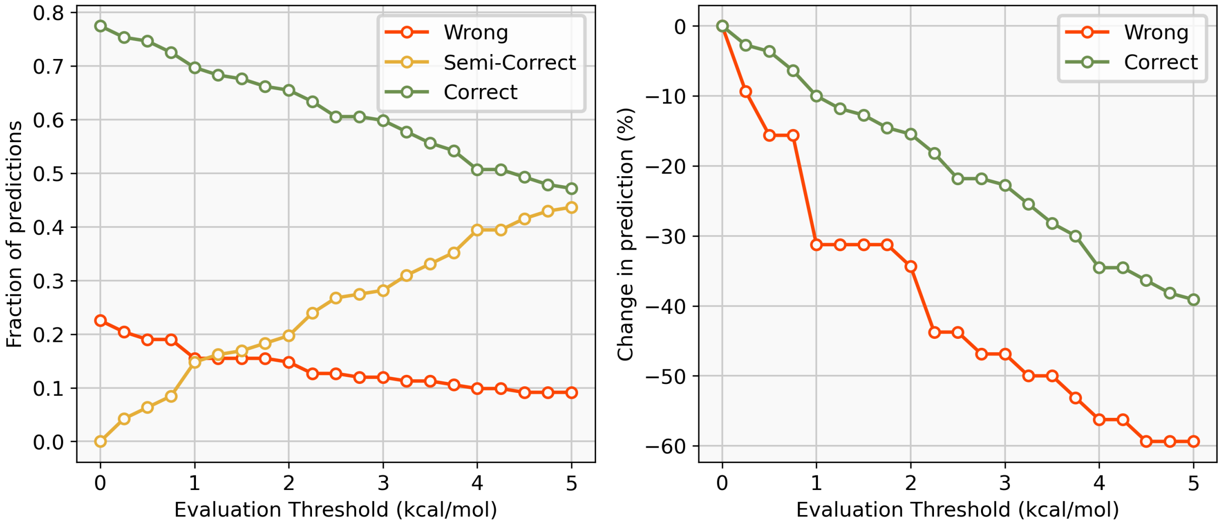

Figure S1: Left: Fraction of each prediction class as a function of evaluation threshold. Right: Change of fraction of correct and wrong predictions as a function of evaluation threshold.

Potential complexes for C–H activation of a specific substrate

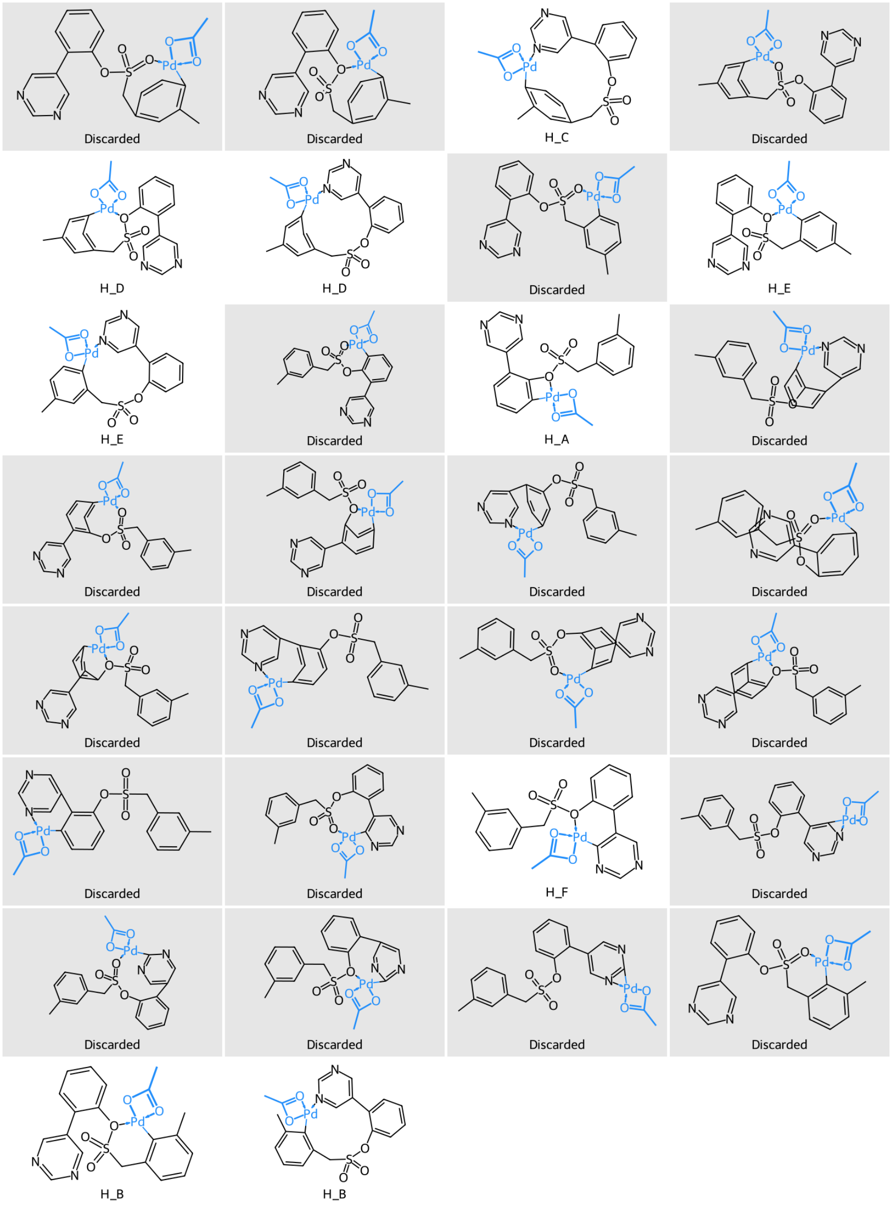

Figure S2: All potential palladacylce intermediates for a substrate from Achar et al. [1]. Via screening of the MMFFs energy contribution from the angle-related terms, unreasonable combinations of C–H site and directing groups are discarded.

Correct regioselectivity prediction for the dataset from Tomberg et al. [2]/Chen et al. [3]

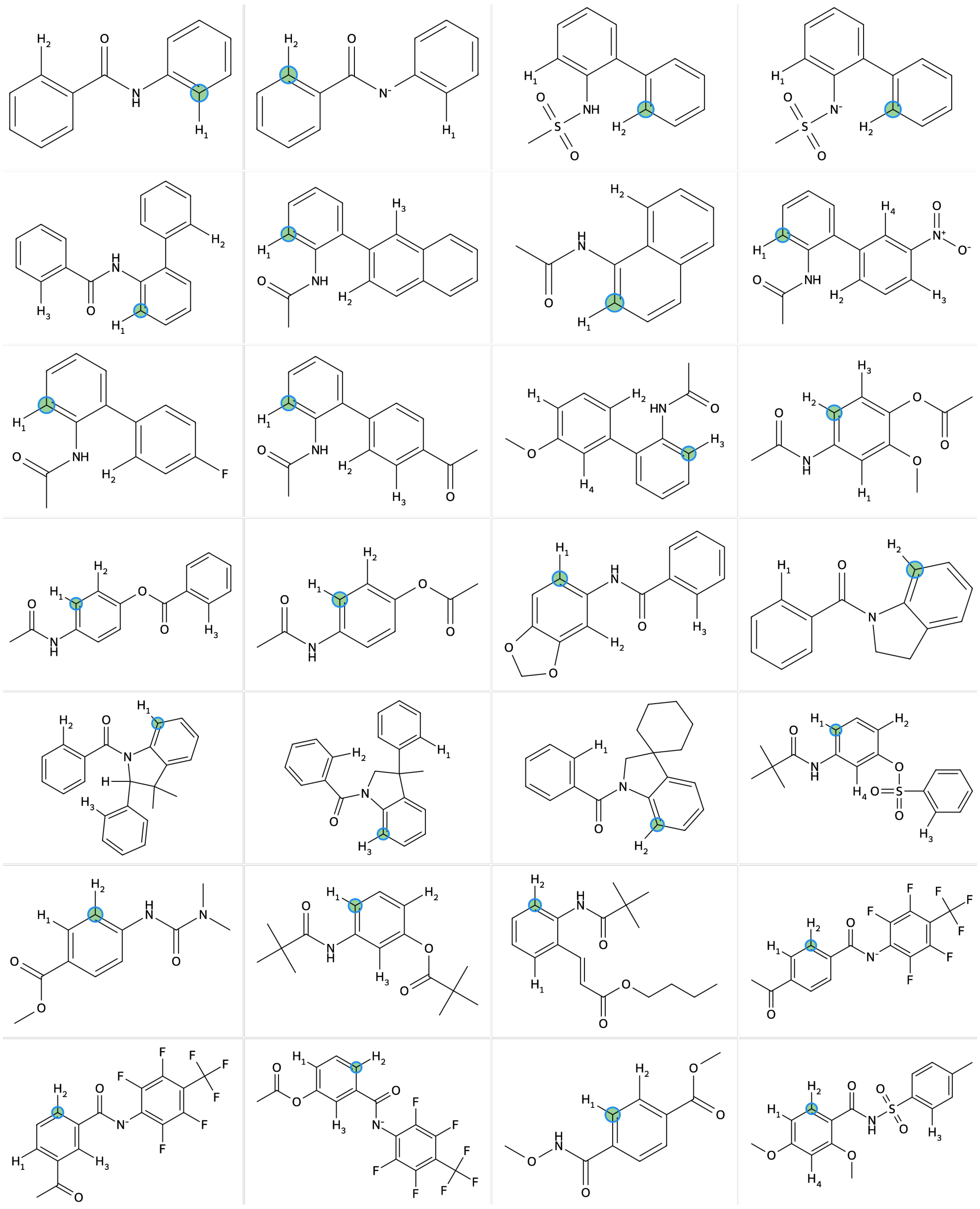

Figure S3: *(1/4)* Correct regioselectivity predictions for molecules from Tomberg et al. [2]/Chen et al. [3]. The predicted reaction site is marked by a blue circle, the experimentally observed reaction site is highlighted with a green circle.

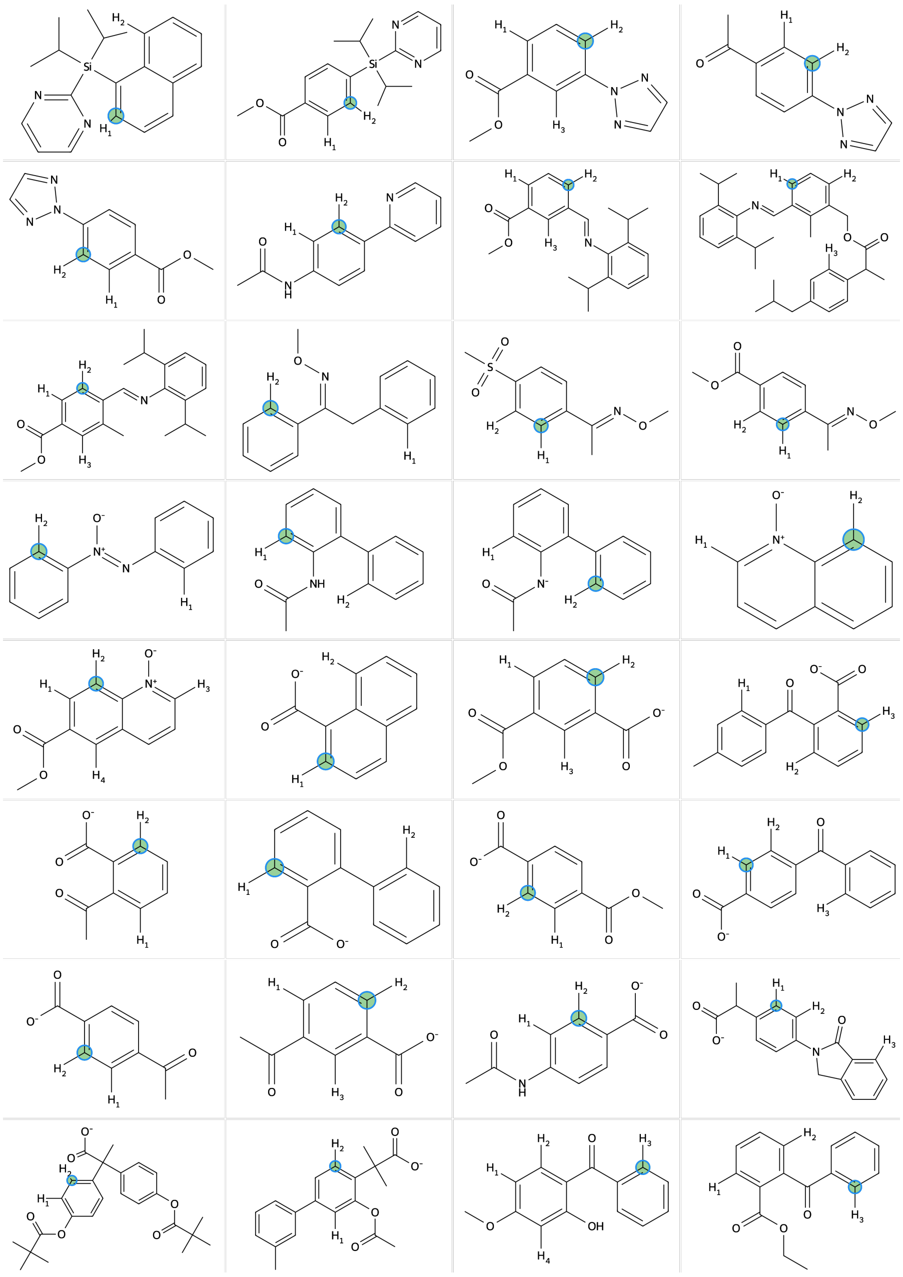

Figure S4: (*2/4*) Correct regioselectivity predictions for molecules from Tomberg et al. [2]/Chen et al. [3]. The predicted reaction site is marked by a blue circle, the experimentally observed reaction site is highlighted with a green circle.

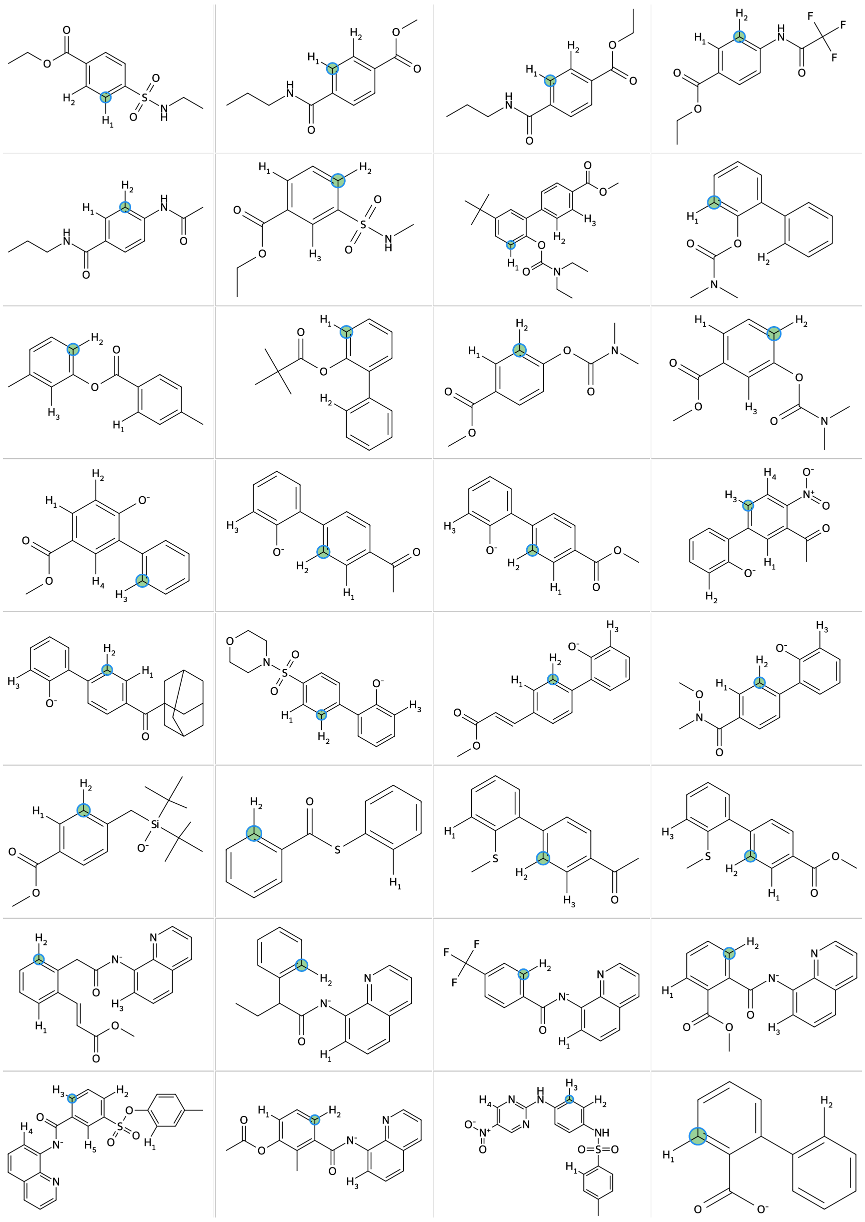

Figure S5: (*3/4*) Correct regioselectivity predictions for molecules from Tomberg et al. [2]/Chen et al. [3]. The predicted reaction site is marked by a blue circle, the experimentally observed reaction site is highlighted with a green circle.

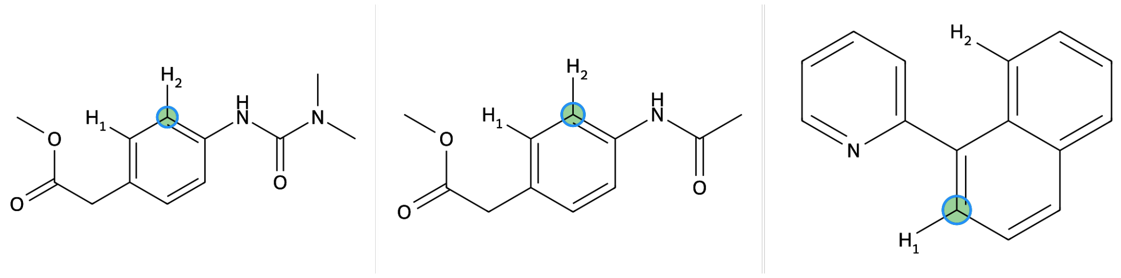

Figure S6: (*4/4*) Correct regioselectivity predictions for molecules from Tomberg et al. [2]/Chen et al. [3]. The predicted reaction site is marked by a blue circle, the experimentally observed reaction site is highlighted with a green circle.

Semi-correct regioselectivity prediction for the dataset from Tomberg et al. [2]/Chen et al. [3]

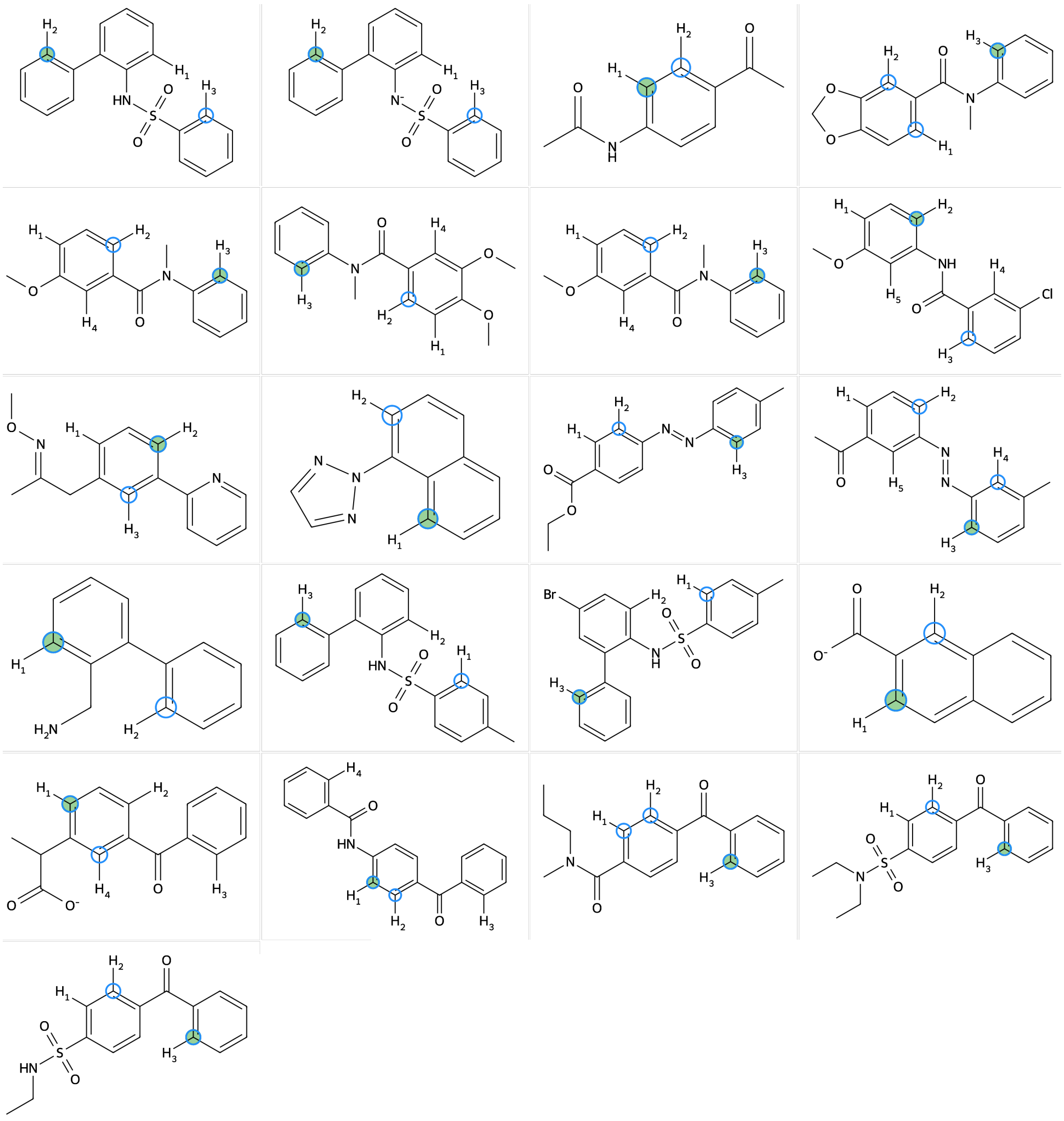

Figure S7: Semi-correct regioselectivity predictions for molecules from Tomberg et al. [2]/Chen et al. [3]. Predicted reaction sites are marked by a blue circle, the experimentally observed reaction site is highlighted with a green circle.

Wrong regioselectivity prediction for the dataset from Tomberg et al. [2]/Chen et al. [3]

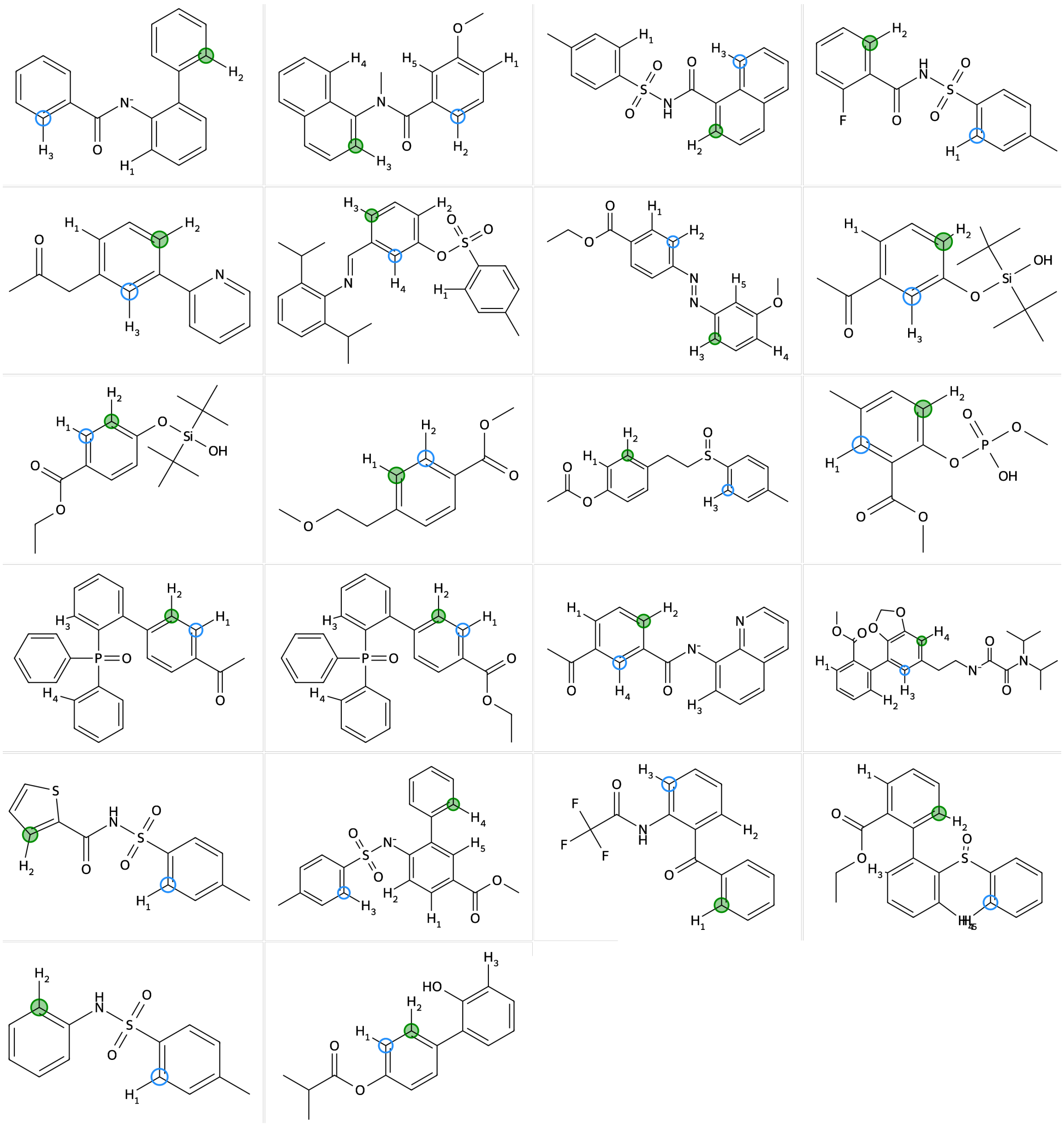

Figure S8: Wrong regioselectivity predictions for molecules from Tomberg et al. [2]/Chen et al. [3]. The predicted reaction site is marked by a blue circle, the experimentally observed reaction site is highlighted with a green circle.

References

1. Achar, T. K.; Zhang, X.; Mondal, R.; Shanavas, M. S.; Maiti, S.; Maity, S.; Pal, N.; Paton, R. S.; Maiti, D. *Angew. Chem., Int. Ed.* **2019**, *58*, 10353–10360. doi:10.1002/anie.201904608.

2. Tomberg, A.; Muratore, M. É.; Johansson, M. J.; Terstiege, I.; Sköld, C.; Norrby, P.-O. *iScience* **2019**, *20*, 373–391. doi:10.1016/j.isci.2019.09.035.

3. Chen, Z.; Wang, B.; Zhang, J.; Yu, W.; Liu, Z.; Zhang, Y. *Org. Chem. Front.* **2015**, *2*, 1107–1295. doi:10.1039/c5qo00004a.
